# GLIS2 promotes colorectal cancer through repressing enhancer activation

**DOI:** 10.1038/s41389-020-0240-1

**Published:** 2020-06-01

**Authors:** Jie Yao, Pin-Ji Lei, Qing-Lan Li, Ji Chen, Shan-Bo Tang, Qiong Xiao, Xiang Lin, Xiang Wang, Lian-Yun Li, Min Wu

**Affiliations:** Frontier Science Center for Immunology and Metabolism, Hubei Key Laboratory of Cell Homeostasis, Hubei Key Laboratory of Developmentally Originated Disease, Hubei Key Laboratory of Enteropathy, College of Life Sciences, Wuhan University, Wuhan, Hubei 430072 China

**Keywords:** Oncogenes, Epigenetics

## Abstract

Gene transcription is coordinately regulated by multiple transcription factors. However, a systematic approach is still lacking to identify co-regulators for transcription factors. Here, we performed ChIP-Seq analysis and predicted the regulators for p53-mediated transcription process, from which we confirmed the roles of GLIS2, MAZ and MEF2A in regulating p53 target genes. We revealed that GLIS2 selectively regulates the transcription of *PUMA* but not *p21*. GLIS2 deficiency caused the elevation of H3K27ac and p53 binding on the *PUMA* enhancer, and promoted *PUMA* expression. It increased the rate of apoptosis, but not cell cycle. Moreover, GLIS2 represses H3K27ac level on enhancers, regulates the gene expression related with focal adhesion and promotes cell migration, through inhibiting p300. Big data analysis supports *GLIS2* as an oncogene in colon cancer, and perhaps other cancers. Taken together, we have predicted candidates for p53 transcriptional regulators, and provided evidence for *GLIS2* as an oncogene through repressing enhancer activation.

## Background

Transcription factors binding to enhancers is one of the critical steps in transcription activation. The DNA binding motifs of transcription factors are critical for recognition of enhancer sequences. Recently, the development of epigenomics revealed the important roles of enhancer activation and silencing in the regulation for the downstream genes of signaling pathways^1–5^.

Epigenetic marks on chromatin are important signatures for cell identification, which co-operate with transcription factors to regulate transcription^1,6,7^. Several histone modifications act as critical marks for enhancer activities. H3K4me1 is the mark for enhancer priming^6,8^; H3K27ac for active enhancers and H3K27me3 for silent enhancers^5^. Though the initial discovery was made with ChIP-Seq of mediator subunits, now H3K27ac in the intergenic chromatin is widely used for identification of active enhancers^7,9,10^. Therefore, whether the enhancer activity is regulated has become one of the emerged question for both signaling transduction and epigenetic fields.

The tumor protein p53 is the most extensively studied transcription factor in mammals^11–14^. p53 is activated upon DNA damage or oncogene activation. It exerts its function through transcription activation of tumor suppressors, such as cyclin-dependent kinase inhibitor 1A (*p21/CDKN1A*) to arrest cell cycle and BCL2 binding component 3 (*PUMA/BBC3*) to induce apoptosis^11,12^. MDM2 proto-oncogene (MDM2), a ubiquitin E3 ligase and a p53 target gene, interacts with p53 and promotes its degradation, which forms a negative feedback loop to control p53 protein level in normal cells^11,12,15^. Till now, more than 100 genes were experimentally proved, and thousands were predicted as p53 target genes^11^. The accumulating results indicated that p53-activated genes varies in different cell lines, which raises the questions how these genes are selectively regulated in different cell lines, an open question in the transcription field for many years^11,12^.

Selective transcription is regulated by many factors, which act synergistically according to the upstream signaling. With the development of functional genomics, the genome distribution profiles of many transcription factors, such as p53, have been determined^16–19^, however, the utilization of these genome-wide profiling data is quite limited. Here, we performed p53 ChIP-Seq analysis upon two different stimuli, and predicted transcription regulators of p53-mediated transcription. We experimentally proved GLIS family zinc finger 2 (GLIS2) as a repressor for p53 target apoptotic gene *PUMA*. *GLIS2* has been proposed as an oncogenic gene in leukemia, caused by the fusion of *GLIS2* with *CBFA2T3* which led to *GLIS2* overexpression^20–22^. GLIS2 mutation has been shown to be related with nephronophthisis in human and mice^23^. But whether GLIS2 functions in other types of cancers and the underlying molecular mechanisms are not determined. Our ChIP-Seq data, together with transcriptome and enhancer analysis, indicated a role for GLIS2 in regulating enhancer activity, probably through repressing the expression of E1A binding protein p300 (p300).

## Materials and methods

### Study design

The aim was to reveal the role and molecular mechanism for GLIS2 in promoting colorectal cancer. From a ChIP-Seq analysis carried out for p53, H3K27ac, and p300 in HCT116 cells, we found novel transcription regulators for p53 target genes. The candidates were then validated with siRNA knockdown and quantitative PCR of p53 target genes. GLIS2 was selected from the three confirmed genes was selected and the molecular mechanism was studied. The function for GLIS2 in colorectal cancer was studied with cell and animal models, and The correlation between GLIS2 and cancers were further analyzed with online big data. For all the deep sequencing analysis, two biological replicates were studied; and for all the other experiments, at least three biological replicates were studied.

### Reagents and cell lines

Antibodies recognizing GLIS2 (LSBio LS-C336253, Thermo PA5-40314), β-Actin (Abclonal AC004), MDM2 (Abcam ab3110), p53 (CTS 2527, Santa Cruz sc-126), P-p53(15S) (CST 9286), HA (Abcam ab9110), PUMA (CST 4976), Halo (Promega G921A), Flag (Sigma F1804), p300 (Abcam ab14984), H3K27ac (Abcam ab4729), H3K4me1 (CST 5326), H3K4me3 (Millipore 04-745), p21 (CST 2947), CHK2 (Epitomics 3428), GAPDH (Abclonal AC002), and LMNB1 (Abcam ab16048) were purchased from indicated commercial sources. Dynabeads MyOne streptavidin C1 were from Thermo-Fisher. Protein G-Sepharose beads were from GE Healthcare. PCR primers were custom synthesized by BGI and siRNAs by GenePharma. Nutlin-3a was purchased from Selleck and 5-FU from Sigma. HCT116, HL7702 and HepG2 Cell lines were purchased from Cell Bank of Chinese Academy. A549 and HeLa were purchased from ATCC. All the cell lines were cultured under recommended conditions according to the manufacturer’s instruction with 10% FBS.

### Reverse transcription and quantitative PCR

Cells were scraped down and collected with centrifugation. Total RNA was extracted with RNA extraction kit (Aidlab) according to the manufacturer’s manual. Approximately 1 μg of total RNA was used for reverse transcription with a first strand cDNA synthesis kit (Toyobo). The resulted cDNA was then assayed with quantitative PCR. β-actin was used for normalization. The sequences of primers are in Supplementary Table 1. Assays were repeated at least three times. Data were shown as average values ± SD of at least three representative experiments. *P*-value was calculated using student’s *t-*test.

### Cell fractionation

Cells were harvested and spun down in cold PBS. Ten volumes of buffer A (10 mM Tris-HCl pH 7.4, 5 mM MgCl_2_, 10 mM NaCl, 1 mM DTT, proteinase inhibitors) was added to the cells, and incubated on ice for 10 min. Then 0.5 volumes of buffer B (10 mM Tris-HCl pH 7.4, 5 mM MgCl_2_, 10 mM NaCl, 1 mM DTT, proteinase inhibitors, 10% NP-40) was added to the cells, and incubated on ice for 1 min. The cell suspension was vortexed for 5 s and centrifuged at 800 × *g* for 5 min at 4 °C. The supernatant was collected as cytoplasm fraction. The above steps were repeated once more and the supernatant was discarded. The sediment was suspended in 10 volumes of PBS as the nuclear fraction. SDS loading buffer was added to the cell fractions for western blotting.

### Immunofluorescent staining

Cells were cultured on coverslips and fixed with freezing methanol after washing twice in PBS. The coverslips were then washed three times by PBS and blocked in PBS with 1% BSA for 10 min. The coverslips were hybridized with primary and secondary antibodies for 1 h each. Then the coverslips were mounted with prolong anti-fade kit (Invitrogen) and observed with fluorescent microscopy.

### ChIP assay

ChIP assay was performed as previously described^24^. Briefly, ~1 × 10^7^ cells were cross-linked with 1% formaldehyde for 10 min, and quenched with 0.125 M glycine for 5 min. Cells were then washed three times with PBS and harvested in ChIP lysis buffer (50 mM Tris-HCl, pH 7.6, 1 mM CaCl_2_, 0.2% Triton X-100). DNA was digested to 150–300 bp by MNase (for histone modifications) or sonicated to 200–500 bp (for transcription factors) before extensive centrifugation. Four volume of ChIP dilution buffer (20 mM Tris-HCl, pH 8.0, 150 mM NaCl, 2 mM EDTA, 1% Triton X-100, 0.1% SDS) was added to the supernatant. The resulted lysate was then incubated with protein G beads and antibodies at 4 °C overnight. The beads were washed five times and DNA was eluted by ChIP elution buffer (0.1 M NaHCO_3_, 1% SDS, 20 µg/ml proteinase K). The elution was incubated at 65 °C overnight and DNA was extracted with DNA purification kit (TIANGEN). The purified DNA was assayed by quantitative PCR. Assays were repeated at least three times. Data were shown as average values ± SD of at least three representative experiments and *p-*value was calculated using student’s *t-*test. The sequences of primers are in Supplementary Table 1.

### Capture-ChIP

In total, 1 × 10^7^ FB-EGFP or FB-GLIS2 stable cells were harvested, cross-linked with 1% formaldehyde for 10 min, and quenched with 0.125 M glycine for 5 min. Cells were lysed in 1 mL RIPA buffer (10 mM Tris-HCl, 1 mM EDTA, 0.1% sodium deoxycholate, 0.1% SDS, 1% Triton X-100, pH 8.0), and rotated for 15 min at 4 °C. Cell lysates were centrifuged at 2300 × *g* for 5 min at 4 °C to isolate the nuclei. Nuclei were suspended in 500 μl of 0.5% SDS lysis buffer (0.5% SDS, 10 mM EDTA, 50 mM Tris-HCl, pH 8.0) and subjected for sonication to shear chromatin fragments to an average size between 200 and 500 bp. Fragmented chromatin was centrifuged at 16,100 × *g* for 10 min at 4 °C. Four hundred and fifty milliliter of supernatant was transferred to a new Eppendorf tube and NaCl solution was added to the final concentration of 300 mM. Supernatant was then incubated with 10 μl of Dynabeads MyOne streptavidin C1 (Thermo-Fisher Scientific) at 4 °C overnight. Then the Dynabeads were washed twice with 2% SDS, twice with RIPA buffer containg 0.5 M NaCl, twice with LiCl buffer (250 mM LiCl, 0.5% NP-40, 0.5% sodium deoxycholate, 1 mM EDTA and 10 mM Tris-HCl, pH 8.0), and twice with TE buffer (10 mM Tris-HCl, 1 mM EDTA, pH 8.0). The chromatin was eluted in SDS elution buffer (1% SDS, 10 mM EDTA, 50 mM Tris-HCl, pH 8.0) followed by incubation at 65 °C overnight. The DNA was treated with RNase A (5 mg/ml) and protease K (0.2 mg/ml) at 37 °C for 30 min, and extracted with DNA purification kit (TIANGEN). The purified DNA was assayed with quantitative PCR or subjected for library construction.

### Cell cycle analysis with flow cytometry

Cells were harvested after digestion with 0.05% Trypsin-EDTA. The cells were then washed twice with PBS and fixed in ice-cold 70% ethanol overnight. Fixed cells were washed twice with PBS and stained in PBS containing propidium iodide (PI, 50 μg/mL) and RNase (100 μg/mL) for 30 min at 37 °C. Cell cycle analysis was performed on an Epics XL-MCL flow cytometer (Beckman Coulter) with System II (version 3.0) software (Beckman Coulter).

### Cell viability assay

HCT116 cells were transfected with the indicated siRNAs for 12 h in 6-well plates. Then cells were seeded on the 96-well plate around 3000 cells per well, incubation for 24, 48, and 72 h respectively. Then the cells were added with 5 μl MTT (5 μg/μl) each well and incubated for 4 h at 37 °C. Then cells were added with 100 μl lysate buffer (50% DMF + 30% SDS, pH 4.7) each well and incubated for 4 h. Signals were collected by Microplate System. Assays were repeated at least three times. Data were shown as average values ± SD of at least three representative experiments and *p-*value was calculated using student’s *t-*test.

### Real-time cell analysis (RTCA) of cell proliferation

Cell proliferation was analyzed with RTCA instrument (ACEA Bioscience Inc.) according to the manufacturer’s instruction as described before^25^. Cells were cultured at 10,000 per well in CIM-Plate wells. The cell index signals were read by xCELLigence RTCA DP Analyzer (ACEA Bioscience Inc.).

### Transwell invasion assay

In total, 1 × 10^5^ HCT116 cells were plated in medium without serum or growth factors in the upper chamber with a Matrigel-coated membrane (24-well insert; pore size, 8 µm; BD Biosciences), and medium supplemented with 10% fetal bovine serum was used as a chemoattractant in the lower chamber. After 36 h of incubation, cells that did not invade through the membrane were removed by a cotton swab. Cells on the lower surface of the membrane were stained with crystal violet and counted. Assays were repeated at least three times. Data were shown as average values ± SD of at least three representative experiments and *p-*value was calculated using student’s *t-*test.

### Xenograft experiments in mice

The 5-week-old male BALB/C nude mice were purchased from Beijing HFK Bioscience Co. Ltd. Colon cancer model was established by injecting subcutaneously 8 × 10^5^ HCT116 cells per site into the flank regions of the mice. Tumor volumes were measured twice a week using calipers. Tumor volumes were calculated as *V* = 0.5 × length × width^2^. After 24 days of injection, the tumors were harvested and weighed.

### Pipeline of RNA-seq data analysis

RNA-seq library was constructed by using Illumina TruSeq library construction kit. Five micrograms of total RNA was used for each sample according to the manufacturer’s instruction. The libraries were sequenced using HiSeq X Ten for 100 bp paired-end sequencing. Quality control of mRNA-seq data was performed using Fatsqc and low-quality bases were trimmed. All RNA-seq data were mapped to the human genome (hg19) by TopHat (version 2.1.1) and allow maximum 2 mismatch. The gene expression level was calculated by Cufflinks with default parameters and gene ontology analysis was performed using DAVID (https://david.ncifcrf.gov).

### Pipeline of ChIP-Seq data analysis

For ChIP-Seq analysis, Fastqc was used for raw data quality control. Cutadapt was used to remove law quality bases and library adaptor contamination (cutadapt –a AGATCGGAAGAGCACACGTCTGAACTCCAGTCAC -A AGATCGGAAGAGCGTCGTGTAGGGAAAGAGTGT -m 20). After data filter, quality control of clean reads was performed by Fastqc again.

Bowtie2 (bowtie2 -q--phred33--end-to-end--sensitive -k 1 -p 5--fr--no-mixed--no-discordant -X 1000) was used for data mapping to human reference genome hg38^26^. Samtools was used to sort BAM file and filter duplicate reads^27^. Only unique mapped reads were accepted for further analysis. MACS1.4 was used for ChIP-Seq peaks calling with band width 500, model fold ranges from 5 to 50, *p-*value cutoff 1e10−8^28^. Then HOMER annotatePeaks.pl was used to annotate ChIP-Seq peaks compared with reference genome hg38. AME in MEME Suite was used to find out significant enriched motif and Deeptools was used to display ChIP-Seq signal around TSS sites and peaks binding sites^29^.

To predict transcriptional co-regulators for p53, the 200 bp DNA sequences around p53 peak summit sites were used for AME analysis, ame --verbose 1 --oc ame-output-dir --control DMSO_P53_summits_200bp.fasta --bgformat 1 --scoring avg --method ranksum --pvalue-report-threshold 0.05 5Fu_P53_summits_200bp.fasta HOCOMOCOv11_full_HUMAN_mono_meme_format.meme. Then the motifs from 5-Fu or Nutlin-3a flank sequences were further compared with DMSO to filter the conserved and bias sites.

### Identification of typical and super enhancers

Enhancers were identified by the algorithm developed by Richard A. Young^7^. Briefly, significant distal H3K27ac peaks (peak boundary 1.5 kb or peak center 3 kb away from gene TSS) were identified, and the peaks whose distance was shorter than 12.5 kb were merged together. The enhancers were ranked by total signal of H3K27ac, and a plot was drawn to show the increased H3K27ac signal. Then a tangent line with slope 1 was found of the curve and the intersection point was determined as the tangency point. Enhancers above the tangency point were defined as super enhancers, meanwhile enhancers below it were defined as typical enhancers.

### Survival analysis

The disease-free survival (DFS, also called relapse-free survival and RFS) and overall survival analysis were carried out via GEPIA performs (http://gepia.cancer-pku.cn/). GEPIA uses log-rank test, also called the Mantel–Cox test, for the hypothesis evaluation. The cox proportional hazard ratio is based on Cox PH Model. The datasets of colon cancer are based on TCGA-COAD (Colon adenocarcinoma).

### Statistical analysis

RNA-seq data of colon cancer tissues, and survival information of patients in TCGA (The Cancer Genome Atlas) project were downloaded from the Genomic Data Commons (GDC) portal site (https://portal.gdc.cancer.gov/)^30^. Overall survival (OS), progression-free survival (PFS), and disease-free survival (DFS) were evaluated by Kaplan–Meier survival analysis and log-rank test. On the basis of the *GLIS2* expression level, cases were divided into two groups according to previously described methods^31,32^.

## Results

### Genome-wide analysis of p53 ChIP-Seq upon different stimuli

**Fig. 1:**
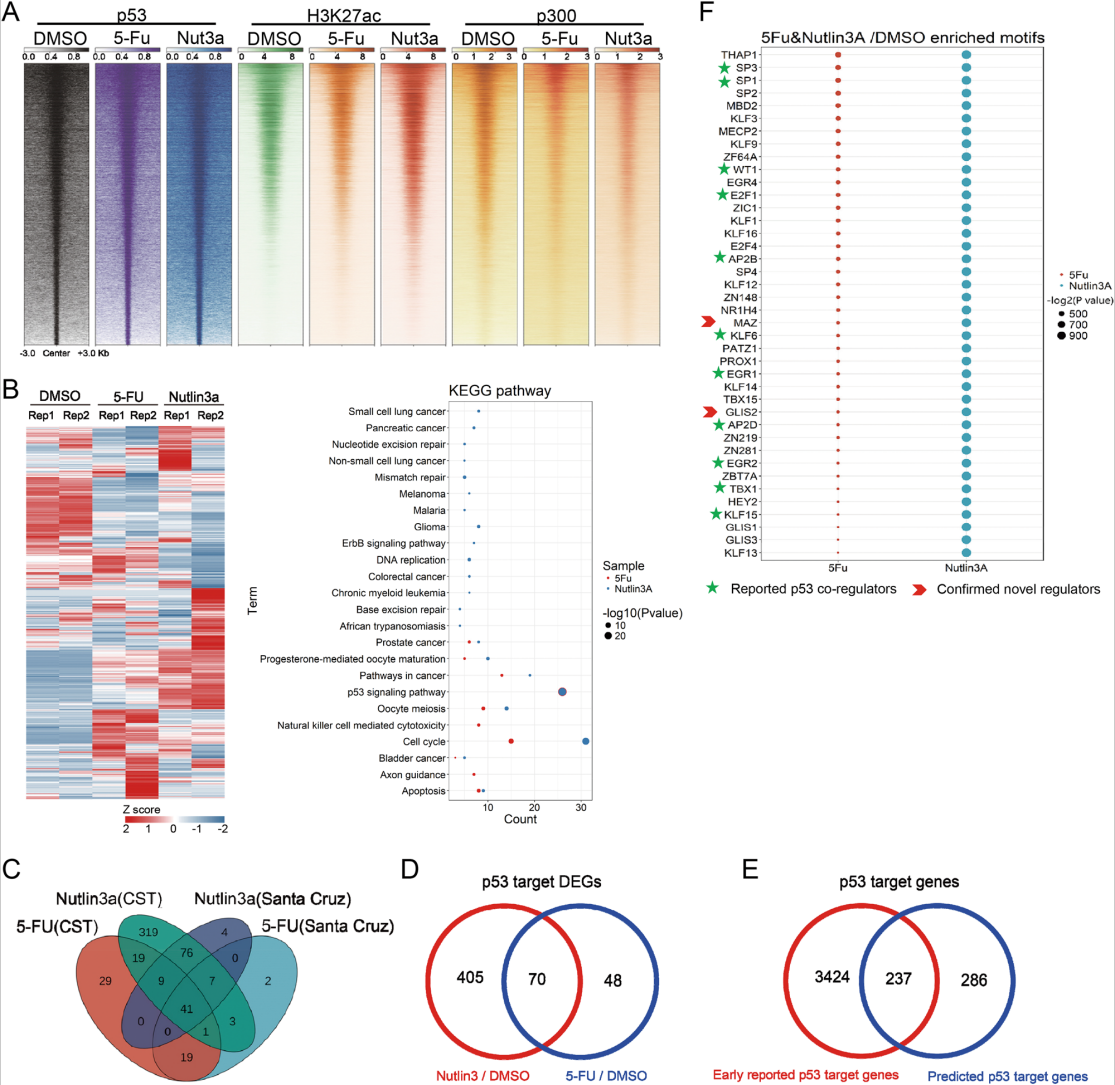
Multidimensional analysis of the p53 transcriptional program. **a** Heat maps generated from ChIP-seq data showing the occupancy of p53, H3K27ac, and p300 in HCT116. All rows are centered on the p53 peaks. **b** Heatmap showing the differential expression genes under DMSO, 5-Fu and Nutlin3A treatment (left) and KEGG pathway enrichment analysis of differential expression genes (right). **c** Venn Diagram analysis on p53 binding genes with two different p53 antibodies. **d** Venn Diagram shows the number of p53 target genes, Nutlin3A compares to DMSO and 5-Fu compares to DMSO. **e** Venn Diagram shows the number of potential p53 targets (right) compared with the number of datasets that commonly identify them (left). **f** A list of p53 co-regulators predicted with indicated method. The green stars point out the reported p53 co-regulators for the transcription of p53 target genes. The red arrows point out the experimental verified genes in our study.

To investigate the distribution of p53 binding on chromatin upon different stimuli, we used two small molecule chemicals to activate p53 in HCT116 cells. 5-Fluorouracil (5-Fu) is a widely used chemotherapy drug to activate p53 signaling pathway. Nutlin-3a is an inhibitor of p53-MDM2 interaction and can activate the expression of p53 target genes. The activation of p53 target genes *p21*, *PUMA*, and *MDM2* was confirmed before further study (Supplementary Fig. S1A & B). ChIP-Seq studies of H3K27ac and p300 were performed at the same time. The results showed that p53 binding was nicely correlated with H3K27ac and p300 level on chromatin (Fig. 1a), suggesting all these p53 binding sites may have potential enhancer activities. The results of RNA-Seq revealed that the different expressed genes (DEGs) were mostly enriched in p53 signaling pathways, which fit our expectation (Fig. 1b, Sup Tables 2–5). Two antibodies were used for p53 ChIP-Seq. One is from Santa Cruz and the other from CST. We noticed the CST antibody have higher signals and targets more genes on chromatin (Fig. 1c and Supplementary Fig. S1C). Since and heavily modified N terminal of activated p53 might affect antibody recognition and the data from Santa Cruz antibody were mostly covered by that from CST antibody, we used the data from CST antibody for further analysis. The consensus motif for p53 binding was predicted, which was almost identical with that in public database, indicating our analysis was well performed (Supplementary Fig. S1D). Moreover, Nutlin-3a treatment led to identification of more p53 binding sites (Supplementary Fig. S1C), suggesting Nutlin-3a may be more powerful to activate p53 transcription activity than 5-Fu when p53 protein level was comparative (Supplementary Fig. S1B). When looking at the typical p53 target genes in Genome Browser, p53 forms sharp peaks on chromatin, which was nicely overlapped with H3K27ac and p300 peaks (Fig. 1a, Supplementary Fig. S1E). All these indicate our data are reliable.

We overlapped the genes bound by p53 with DEGs upon p53 activation, and identified 475 potential p53 target genes with Nutlin-3a treatment, and 118 p53 target genes responding 5-Fu (Fig. 1d, Supplementary Table 6). Totally 70 genes were overlapped between the two groups (Fig. 1d). The KEGG analysis indicated that both drugs activated the similar number of genes in p53 signaling, while Nutlin-3a activated more genes involved in cell cycle (Fig. 1c). Compared with the previous reported studies^11^, 237 target genes were reported before and 286 were novel p53 target genes (Fig. 1e, Supplementary Table 6).

### Bioinformatic analysis to predict GLIS2’s roles in regulating p53 target genes

To study regulation of p53 transcription, we utilized the 200 bp fragments around p53 peaks summit sites and predicted the potential bound transcription factors on these fragments. The analysis predicted many candidates, among which many have been reported by previous studies (Fig. 1f). We selected 10 unreported genes and performed siRNA knockdown experiments to investigate their effects on the activated expression of p53 target genes. We successfully identified three positive genes, including *GLIS2*, MYC-associated zinc finger protein (*MAZ*), and myocyte enhancer factor 2A (*MEF2A*), which were able to affect the expression of p53 target genes when knockdown (Supplementary Fig. S2). We then noticed that two family members of *GLIS2* were also predicted from the analysis, while *MEF2A* was not shown in the top gene list due to its low *p-*value (Fig. 1f). GLIS2 is a transcription factor containing kruppel-like zinc finger^33–35^. We found one dataset of eGFP-GLIS2 overexpressed in HEK293 cells from ENCODE database (https://www.encodeproject.org/experiments/ENCSR535DIA/), which supported co-localization of GLIS2 with p53 on p53 target genes (data not shown). Then we focused on GLIS2 to study its function in p53 signaling and tumorigenesis.

### Selective repression of p53 target genes by GLIS2

**Fig. 2:**
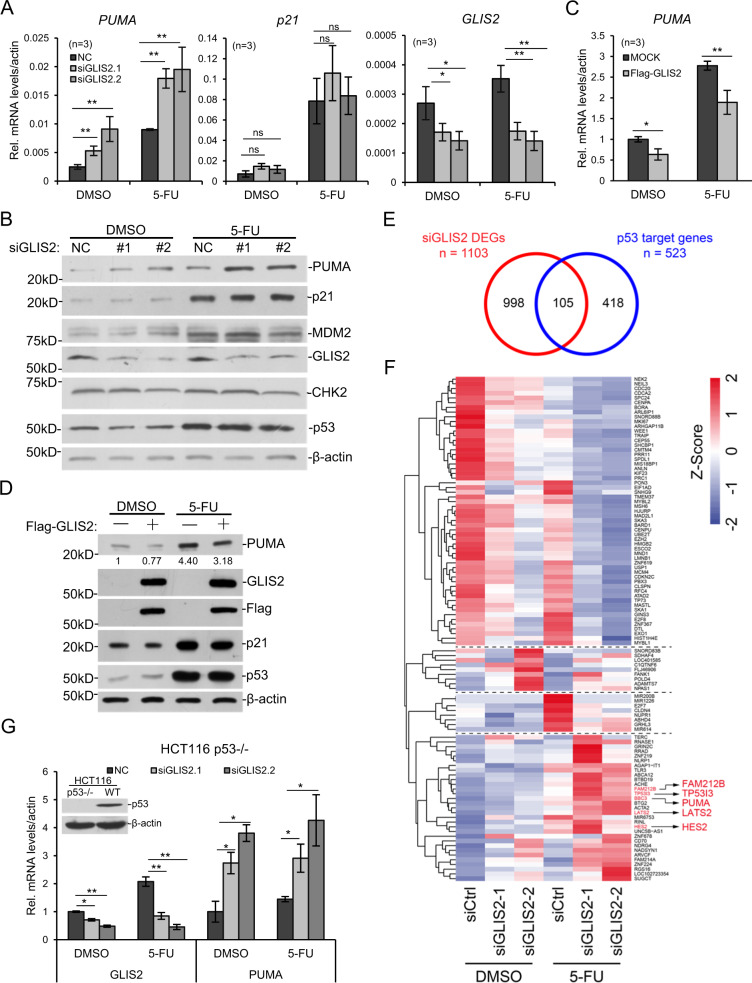
GLIS2 negatively regulates the expression of PUMA. **a** HCT116 cells were transfected with two different siRNAs of GLIS2 for 48 h followed by treatment with 375 μM 5-FU for 8 h, and the expression of PUMA, p21, and TP53 was determined by qRT-PCR. Data are presented as mean ± SEM of three independent experiments. **b** GLIS2 was knocked down with two different siRNAs in HCT116 and cell lysates were immunoblotted with indicated antibodies. **c**, **d** GLIS2 was exogenous expressed in HCT116 and the expression of PUMA was determined by qRT-PCR (**c**) and western blotting (**d**). PUMA protein abundance was quantified by ImageJ. **e** DEGs between control and GLIS2 knockdown under DMSO or 5-Fu conditions were combined as siGLIS2 DEGs. Venn diagram shows the overlapped gene number between siGLIS2 DEGs and p53 target genes. **f** Heatmap of the overlapped genes in (**e**). Genes validated with RT-PCR were in red. **g** HCT116 p53 knockout cells were prepared as in 2 A and the expression of PUMA was determined by qRT-PCR. *p-value ≤ 0.05, **p-value ≤ 0.01 (*t*-test). Histograms are presented as mean ± s.d. of three biological replicates.

*GLIS2* was knocked down with two different siRNAs and 5-Fu was used to treat HCT116 cells. The results indicated that *GLIS2* deficiency elevated the expression of *PUMA*, in both control and 5-Fu treated cells (Fig. 2a and Supplementary Fig. S2A). Meanwhile, it did not significantly affect the expression of *p21* and *MDM2* (Fig. 2a and Supplementary Fig. S2A). Western blotting also agreed with the RT-PCR result that PUMA protein increased after *GLIS2* knockdown (Fig. 2b). A Flag-tagged GLIS2 was overexpressed in HCT116 cells, and RT-PCR showed that *PUMA* mRNA level was significantly repressed, both in control and 5-Fu treated samples (Fig. 2c), which was also confirmed with western blotting (Fig. 2d). When GLIS2 was overexpressed, we observed that *p21* was downregulated a little bit, at mRNA and protein level (Fig. 2d). It was probably the artificial effect caused by exogenous expression. Similar experiment was performed with Halo-tagged GLIS2 and similar result was observed (Supplementary Fig. S3A).

RNA-Seq analysis was performed to study the function of GLIS2 on the global transcription. Totally 1103 DEGs were identified after GLIS2 knockdown, among which 105 genes were overlapped with the p53 target genes (Fig. 2e, Supplementary Fig. S3B, Supplementary Tables 7 and 8). The relative gene expression levels of the overlapped genes were shown (Fig. 2f). The data also supported that the expression of *PUMA*, but not *p21*, was elevated after GLIS2 knockdown (Fig. 2f). The results for other p53 target genes were confirmed with quantitative RT-PCR (Supplementary Fig. S3C).

To further check GLIS2’s function on *PUMA* expression, we investigated four other cell lines, including one lung cancer cell line, A549, one cervix cancer cell line, HeLa, and two liver cell lines, HL7702 and HepG2. When GLIS2 was knocked down, *PUMA* expression was elevated in all tested cell lines (Sup Fig. S3D). Interestingly, p53 deficiency seems not to affect GLIS2 function, since in p53^−/−^ cells, *GLIS2* knockdown with siRNAs still elevated *PUMA* expression (Fig. 2g), indicating GLIS2 can function on *PUMA* expression independent of p53.

### GLIS2 binds to PUMA promoter

To investigate the mechanism how GLIS2 regulates *PUMA* expression, we first studied the localization of GLIS2 in the cell. HCT116 cells were separated into cytoplasm and nuclear fractions and western blotting was performed. The results showed that GLIS2 exists in the nucleus but was not detected in cytoplasm (Fig. S4A). Immunostaining of endogenous GLIS2 was performed with two different commercial antibodies. The one from LSBIO showed GLIS2 was localized in nucleus (Fig. S4B); while that from Thermo-Fisher showed GLIS2 both in nucleus and cytoplasm (data not shown). Combined with the cell fractionation result, we thought GLIS2 should be a nuclear protein.

**Fig. 3:**
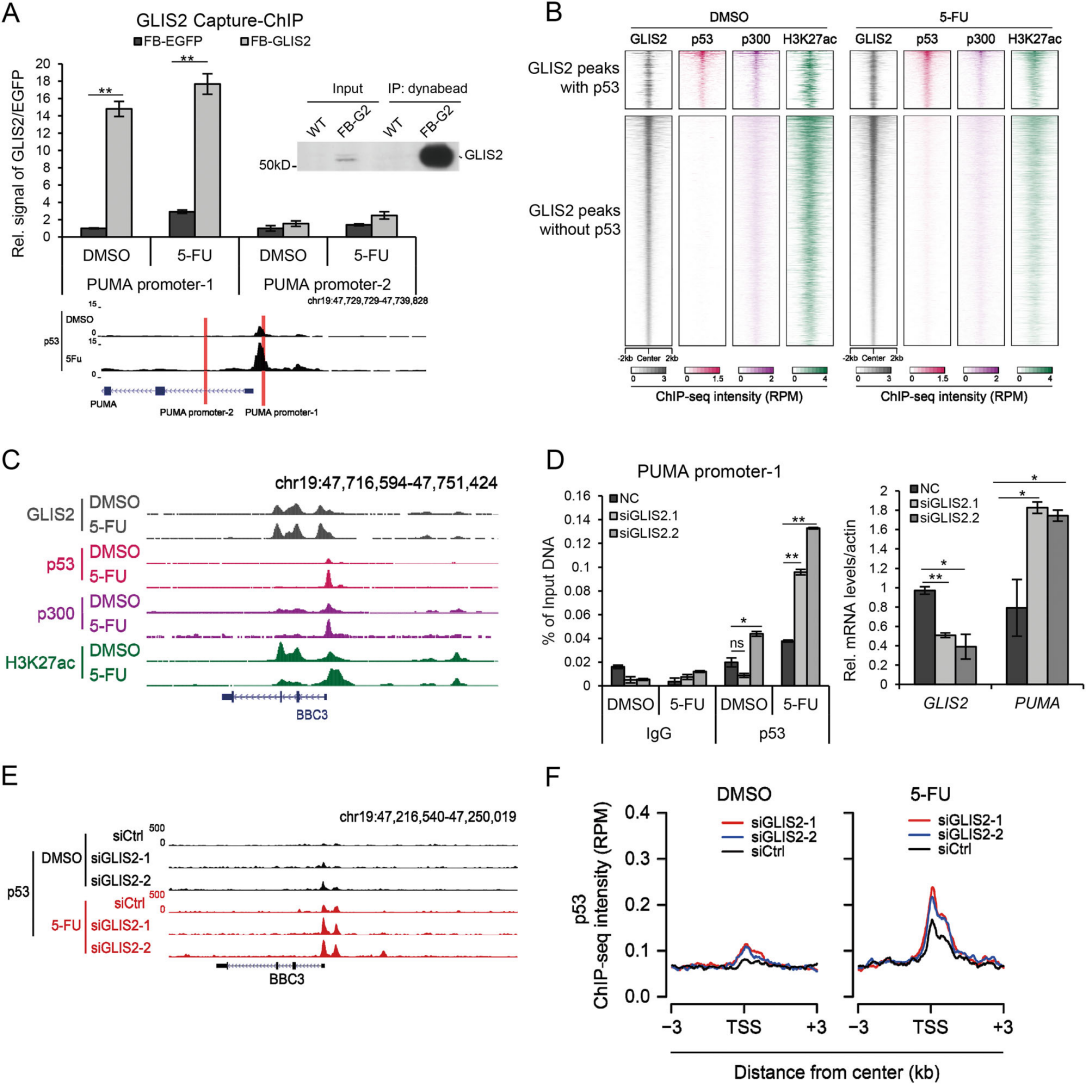
GLIS2 represses p53 binding on PUMA promoter. **a** CAPTURE-ChIP shows GLIS2 binding on PUMA relative to EGFP. The primer sets for qPCR are indicated at the bottom. **b** Heat maps generated from ChIP-seq data showing the occupancy of GLIS2, p53, H3K27ac, and p300 in HCT116. All rows are centered on the GLIS2 peaks. GLIS2 ChIP-seq was performed by Dynabeads MyOne streptavidin C1 (Thermo-Fisher 65001) in FB-GLIS2-expressing HCT116 stable cells. **c** The genome browser view of FB-GLIS2 on PUMA. **d** Cells were prepared as in 2A and ChIP analysis was performed with anti-p53 antibody. **e** The UCSC browser view shows p53 enrichment around PUMA after GLIS2 knockdown. **f** The average signals of p53 enrichment on p53 target genes (*n* = 523). *p-value ≤ 0.05, **p-value ≤ 0.01 (*t*-test). Histograms are presented as mean ± s.d. of three biological replicates.

Next we investigated whether GLIS2 directly binds to PUMA. We constructed a plasmid containing the *GLIS2* cDNA fused with Flag and biotin-acceptor-site (FB) tag, and established a stable cell line with the plasmid in HCT116 cells (Fig. 3a). Capture-ChIP assay was performed and the result indicated FB-GLIS2 directly binds to *PUMA* promoter (Fig. 3b, c). ChIP assays with HA-tagged GLIS2 stable cell line also supported the same conclusion (Supplementary Fig. S4C). DNA pulled down with FB-tagged GLIS2 was assayed with deep sequencing and the result further indicated that GLIS2 is co-localized with p53 on a portion of genes (Fig. 3b), including *BBC3/PUMA* (Fig. 3c). A large number of GLIS2 peaks on chromatin was enriched on gene promoters (Supplementary Fig. S4D), suggesting a role of GLIS2 in regulating transcription.

### *GLIS2* deficiency enhances p53 binding to PUMA

To study whether GLIS2 regulates p53 recruitment to *PUMA*. *GLIS2* was knocked down with two different siRNAs in HCT116, and p53 ChIP assay was performed. The results indicated the bound p53 on *PUMA* significantly increased with GLIS2 deficiency (Fig. 3d). Similar study was performed with a *GLIS2* shRNA stable cell line and supported the same conclusion, and *GLIS2* knockdown seemed to not affect p53 binding on *p21* (Supplementary Fig. S4E, F). ChIP-Seq result further agreed that the bound p53 on *PUMA* increased after *GLIS2* knockdown (Fig. 3e). Further analysis indicated that the average amount of bound p53 on its target genes increased after *GLIS2* knockdown (Fig. 3f). All these results indicated that GLIS2 directly binds to PUMA promoter and represses p53 recruitment.

To study the relationship between p53 and GLIS2, we analyzed all the p53 and GLIS2 binding sites, and defined if the distance between the binding sites of two proteins was <200 bp, then they were considered overlapped (Supplementary Fig. S5A). The genes adjacent to p53 unique sites were enriched in p53 signaling pathways, as expected (Supplementary Fig. S5B). Interestingly, the biological processes and KEGG pathways of the overlapped and p53 unique genes were largely different. Endocytosis was the only common enriched pathway for them (Supplementary Fig. S5B). These suggested the function of genes co-targeted by p53 and GLIS2 is probably distinguished from p53 unique genes. We failed to analyze the genes adjacent to GLIS2 unique sites because there were too many genes and not suitable for analysis. We further overlapped the above adjacent genes with the upregulated and downregulated DEGS after GLIS2 knockdown (Supplementary Fig. S5C, D). Based on the DEG numbers upregulated or downregulated, no significant difference was observed between p53/GLIS2 co-target and GLIS2-only target genes.

### *GLIS2* deficiency elevates H3K27ac level on p53 target genes

**Fig. 4:**
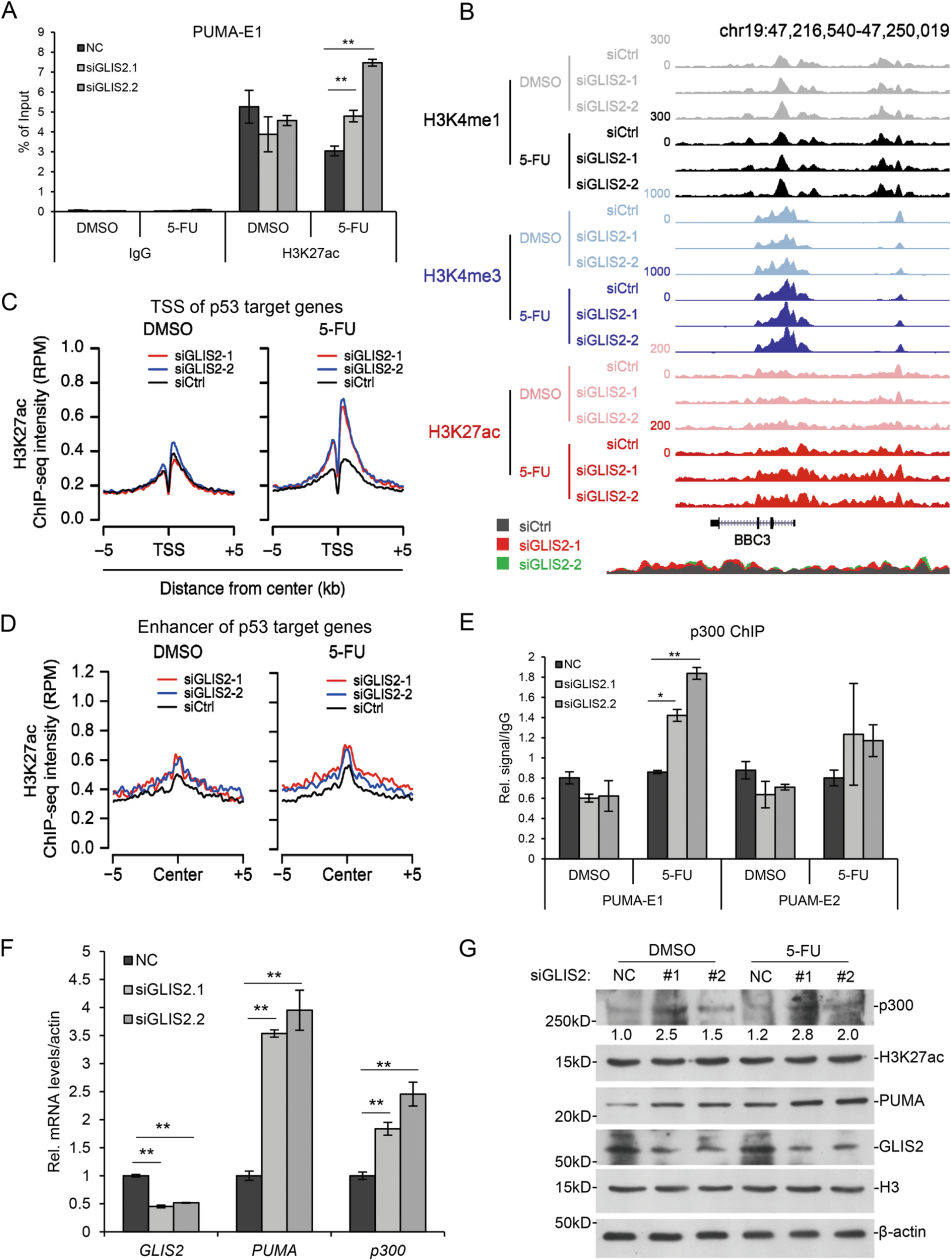
GLIS2 represses enhancer activation of p53 target genes. **a** HCT116 cells were prepared as in 2A. ChIP analysis shows H3K27ac enrichment on PUMA enhancers. **b** The UCSC browser view shows H3K27ac, H3K4me1, and H3K4me3 enrichment around PUMA after GLIS2 knockdown. **c** The average signals of H3K27ac enrichment on p53 target genes. **d** The average signals of H3K27ac enrichment on enhancers of p53 target genes. **e** The average signals of H3K27ac enrichment on enhancers of GLIS2-regulated p53 target genes. **f** ChIP assays to show p300 enrichment on PUMA enhancers after GLIS2 knockdown. *p-value ≤ 0.05, **p-value ≤ 0.01 (*t*-test). Histograms are presented as mean ± s.d. of three biological replicates.

To investigate the mechanism of the repression of p53 recruitment by GLIS2, we first checked the interaction between p53 and GLIS2 with co-immunoprecipitation, but did not get positive result (data not shown). We then examined whether GLIS2 regulates the activity of p53 target enhancers. When *GLIS2* was knocked down, H3K27ac on *PUMA* enhancer significantly increased upon 5-Fu treatment (Fig. 4a). ChIP-Seq analysis were performed and the results showed that GLIS2 deficiency did not affect H3K4me1 on PUMA, but increased H3K27ac around *PUMA* transcription start site (TSS) and enhancer upon 5-Fu treatment (Fig. 4b). The average level of H3K27ac around TSS of p53 target genes increased after *GLIS2* knockdown, especially with 5-Fu treatment (Fig. 4c). H3K27ac on the enhancers of p53 target genes, and those regulated by GLIS2, also increased (Fig. 4d), which indicated that their enhancer activity increased after *GLIS2* knockdown. Further study with ChIP-PCR indicated that p300 bound to *PUMA* enhancers increased after *GLIS2* knockdown (Fig. 4e), suggesting that GLIS2 restricted the amount of p300 on *PUMA* enhancer. Further experiments revealed that upon *GLIS2* knockdown, the mRNA level of *p300* increased (Fig. 4f). Western blotting showed that the protein level of p300 also increased with *GLIS2* deficiency (Fig. 4g). These results indicated that GLIS2 can regulate the global enhancer activity through repressing *p300* transcription.

### *GLIS2* deficiency inhibits cell proliferation and migration, but enhances apoptosis

**Fig. 5:**
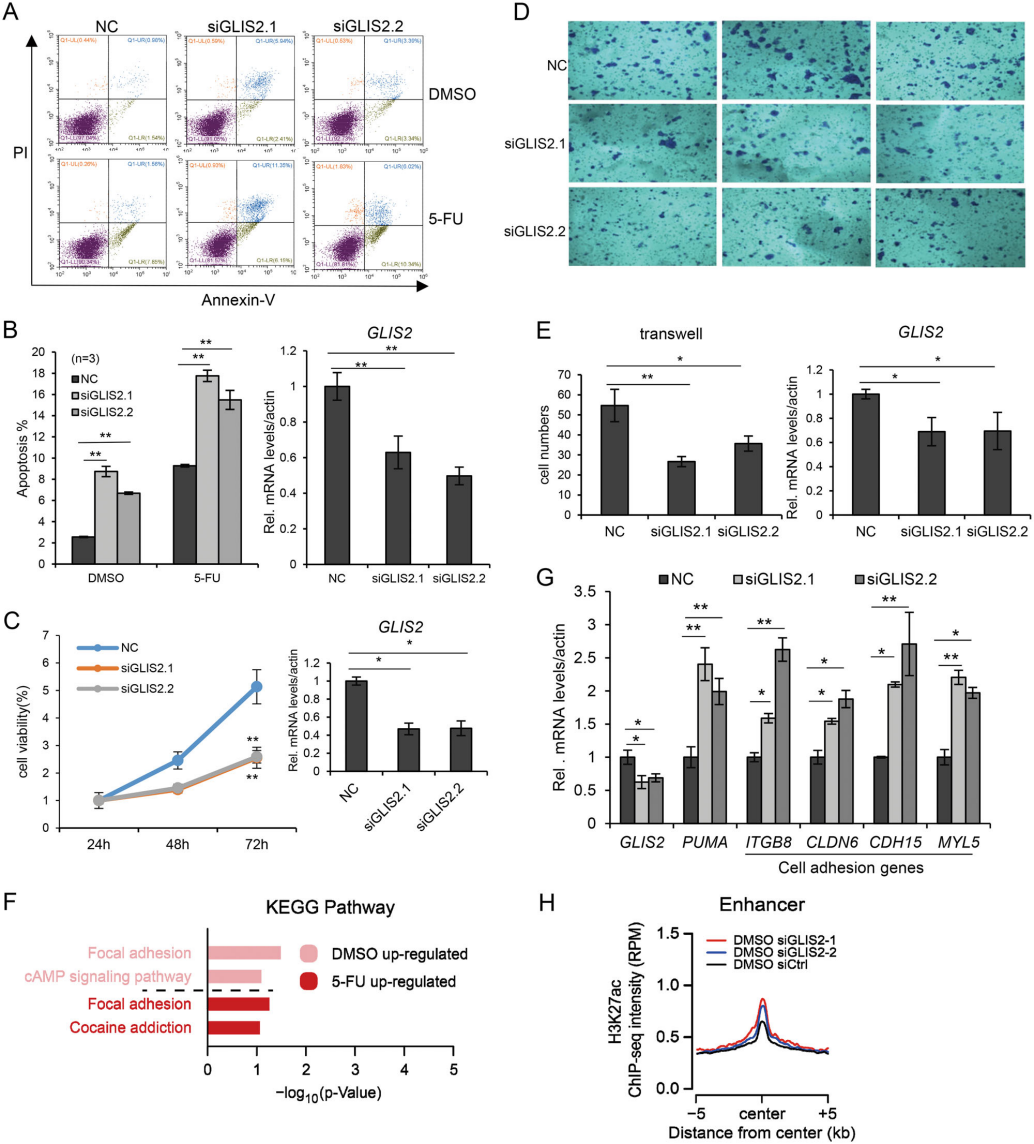
GLIS2 promotes colon cancer cell proliferation and migration. **a**, **b** Cells were prepared as in (**a**). Cells were double stained with annexin-V–FITC and propidium iodide, and then assayed by flow cytometry. The statistical calculation of the proportion of annexin-V-positive cells is shown in (**h**). Transcript levels were determined relative to actin mRNA levels and normalized relative to control cells. **c** MTT assay to show cell proliferation after GLIS2 knockdown. **d**, **e** Transwell assay to show cell migration after GLIS2 knockdown. The statistical calculation of the numbers of migration cells is shown in (**c**). **d** The KEGG pathways enrichment analysis of the GLIS2 repressed DEGs. **e** Validation of the expression of some cell adhesion genes after GLIS2 knockdown by qRT-PCR. **f** The average signals of H3K27ac enrichment on genome-wide enhancers. **g** The expression of PUMA and p300 was determined by qRT-PCR after GLIS2 knockdown. **h** HCT116 cells were prepared as in Fig. 2 a and cell lysates were immunoblotted with indicated antibodies. p300 bands were quantified by ImageJ and normalized to β-actin. *p-value ≤ 0.05, **p-value ≤ 0.01 (*t*-test). Histograms are presented as mean ± s.d. of three biological replicates.

*PUMA* and *p21* are two major target genes for p53, one for apoptosis and the other for cell cycle arrest. When *GLIS2* was knocked down in HCT116 cells, the percentage of apoptotic cell significantly increased (Fig. 5a, b); while the cell percentage of cell cycle phases did not change much (Supplementary Fig. S6A). Exogenous expression of Halo-tagged GLIS2 in HCT116 did not affect cell cycle either (Supplementary Fig. S6B). This further support the selective regulatory function of GLIS2 on *PUMA*, but not *p21*. To further understand the role of GLIS2 in cancer, we studied its function on cell proliferation and migration. MTT assay showed that GLIS2 deficiency significantly inhibits cell proliferation (Fig. 5c). The results of real-time cell analysis (RTCA) also supported that *GLIS2* knockdown caused slower cell proliferation (Supplementary Fig. S6C). The phenotype may be related with the role of GLIS2 in apoptosis. The transwell assay showed that GLIS2 knockdown inhibits cell migration (Fig. 5d, e), suggesting that GLIS2 functions beyond apoptosis regulation.

### GLIS2 represses the expression of genes related with focal adhesion

Since *PUMA*’s function is not related with cell migration, we further investigated our global gene expression data to investigate the mechanism of GLIS2-regulated migration. Since GLIS2 plays a role in repressing transcription, we analyzed the enriched KEGG pathways for the upregulated genes after GLIS2 knockdown in the absence of 5-Fu, which showed that they were involved in focal adhesion (Fig. 5f and Supplementary Fig. S6D). Quantitative RT-PCR verified the function of GLIS2 on some genes involved in focal adhesion, including *ITGB8*, *CLDN6*, *CDH15*, and *MYL5* (Fig. 5g). GLIS2 probably promotes cell migration through regulating the expression of the above genes, which is independent of p53 activation.

### GLIS2 represses enhancers activity globally

The above results suggested that the function of GLIS2 is not restricted to p53 signaling. We studied the effect of GLIS2 on global enhancer activity. Although only a relatively small number of enhancers showed upregulated H3K27ac level (Supplementary Fig. S6E), *GLIS2* knockdown elevated the average H3K27ac level of the total enhancers in the cells (Fig. 5h).

To identify the direct target genes for GLIS2, we overlapped the DEGs after GLIS2 knockdown with the adjacent genes to GLIS2 binding sites and predicted GLIS2 target genes (Supplementary Fig. S7A and Supplementary Tables 9 and 10). Totally 116 upregulated and 50 downregulated GLIS2 target genes were identified. The gene numbers suggested the major function of GLIS2 is to repress transcription, as reported previously. Among these, the upregulated GLIS2 target genes were enriched in apoptosis process and cAMP signaling pathway (Supplementary Fig. S7B), and the downregulated GLIS2 target genes in angiogenesis process.

### *GLIS2* acts as an oncogene in colorectal and other cancers

**Fig. 6:**
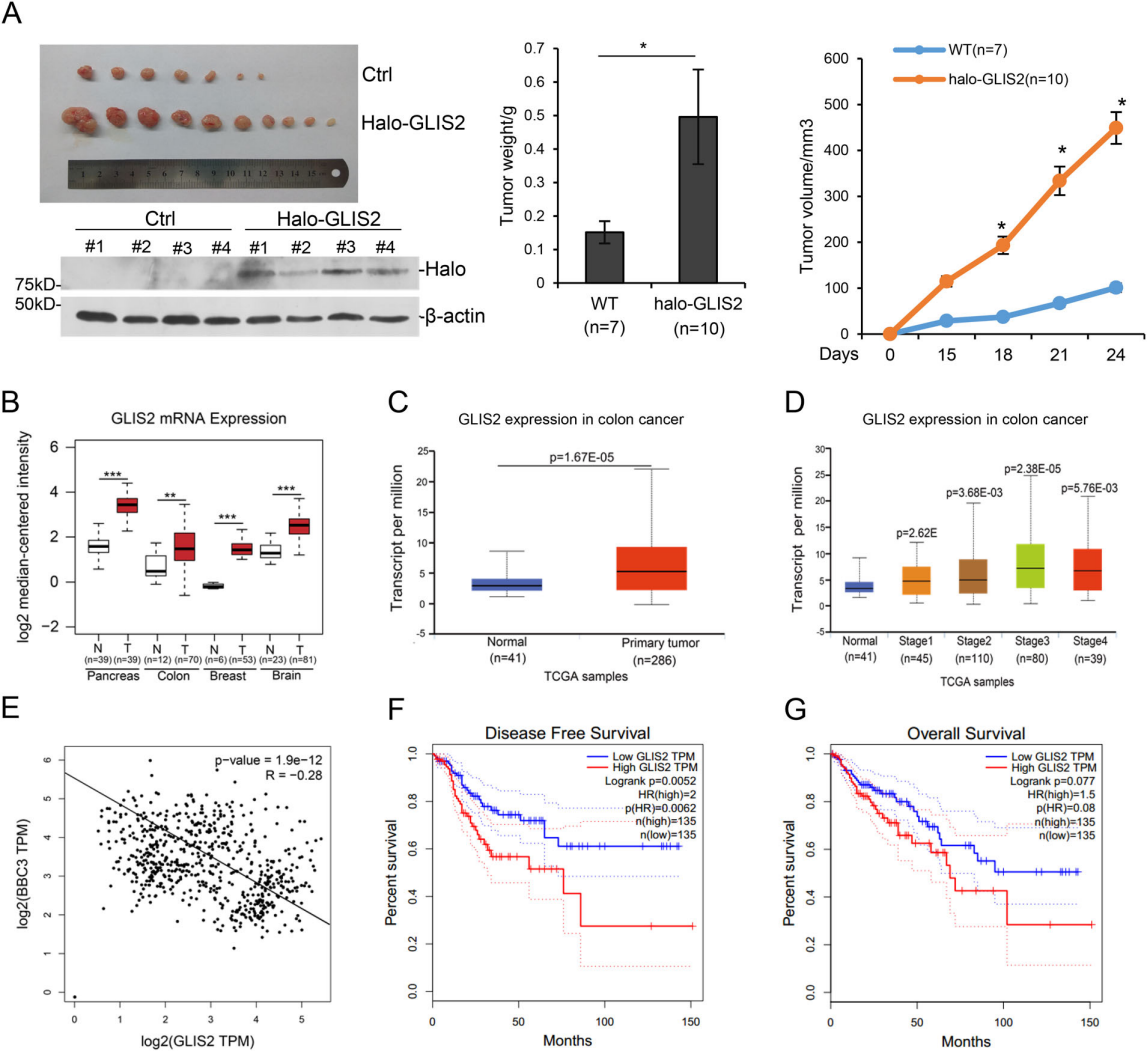
GLIS2 is an oncogene in multiple cancers. **a** Halo-GLIS2 stably expressed cells were injected into nude mice. Tumors were pictured (left). tumor weight (middle) and growth curve (right) were shown as mean ± SEM. Four tumors from each group were random picked and assayed with western blotting to confirm they are from original cell lines. **b** Boxplots show the expression (FPKM) of GLIS2 in normal and cancer tissues. The cancer-related data were downloaded from four independent microarray datasets from the Gene Expression Omnibus database: colorectal carcinoma (GSE9348); invasive breast carcinoma (GSE9014); glioblastoma (GSE4290); pancreatic ductal adenocarcinoma (GSE15471). **c**, **d** The expression of GLIS2 in normal and cancer colon tissues (COAD) was analyzed by an interactive web-portal, UALCAN (http://ualcan.path.uab.edu). **e** The correlation of GLIS2 and BBC3 (PUMA) in normal and cancer colon tissues was analyzed by an interactive web-portal, GEPIA (http://gepia.cancer-pku.cn). **f**, **g** RNA-seq data of colon cancer tissues in TCGA database were analyzed. Overall survival (OS) and disease-free survival (DFS) were analyzed and plotted using the Kaplan–Meier method. The survival rates for patients with high and low GLIS2 expression are plotted as red and blue lines, respectively. The number of patients in each group is shown in parentheses. *p-*Values were calculated using a log-rank test. *p-value ≤ 0.05, **p-value ≤ 0.01 (*t*-test).

To further study the relationship between GLIS2 and tumorigenesis, we injected Halo-tagged GLIS2 stable cells into nude mice. The results showed that GLIS2 expression in HCT116 enhanced the tumor number, weight and volume in xenograft experiment (Fig. 6a). We also tried to establish a knockout or knockdown cell line, but failed to get it after multiple efforts, suggesting GLIS2 was perhaps essential for these cell lines. A pan cancer analysis showed that *GLIS2* exhibited higher expression in pancreas cancer, colon cancer, breast cancer, and brain cancer, in comparison with their corresponding normal tissues (Fig. 6b), suggesting a wide oncogenic role in multiple cancers for GLIS2. Analysis of another online cancer dataset, using an interactive web-portal UALCAN (http://ualcan.path.uab.edu), showed that *GLIS2* is higher expressed in colon cancer compared with the normal tissues (Fig. 6c), and the expression of *GLIS2* is correlated with the tumor TNM stages (Fig. 6d). The expression of *PUMA* is negatively correlated with *GLIS2* expression in colon and cancer tissues, supporting GLIS2’s role in repressing *PUMA* expression (Fig. 6e). Furthermore, low expression of GLIS2 is significantly correlated with the overall survival and disease-free survival rates of colon cancer patients (Fig. 6f, g). To sum up, GLIS2 seems to act as an oncogene and may be useful for the prognosis of colon cancer patients.

## Discussion

In the current study, we applied a bioinformatics approach to identify the potential regulators of p53 target genes. We experimentally verified three novel transcription regulators, including GLIS2, MAZ, and MEF2A. MAZ is an oncogenic transcription factor associated with multiple cancers^36,37^; while MEF2A is tumor repressive and its mutation is associated with tumorigenesis^38,39^. This information will be useful to the study of p53-dependent transcription regulation and the methodology will be useful to study other transcription factors.

*GLIS2* has been proposed as an oncogenic gene in leukemia, in which chromatin translocation causes the fusion of *GLIS2* with *CBFA2T3* and led to *GLIS2* overexpression^20–22^, and its mutation is linked with nephronophthisis in human and mice^23^. But the underlying molecular mechanisms for GLIS2 in these diseases are not determined. Our study showed that *GLIS2* is overexpressed in multiple cancers and may be useful for prognosis of colon cancer. GLIS2 regulates the expression of a subset of p53 target genes. It represses the expression of *PUMA*, but does not affect *p21* and *MDM2*. Correspondingly, *GLIS2* deficiency increased the rate of apoptosis induced by 5-Fu, but did not affect cell cycle distribution. Moreover, GLIS2 regulates the expression of genes related with focal adhesion, which is correlated with its ability of regulating cell migration. Thus, our data indicate that GLIS2 inhibits apoptosis through repressing PUMA expression, and promotes migration through enhancing the gene expression related with focal adhesion, which promotes tumorigenesis.

The regulation of enhancer activity emerged to be one of the critical question to be answered. H3K27ac, the target gene expression level and eRNA amount have been used to evaluate enhancer activity. However, eRNAs of many enhancers are too low to be reliably detected. As comparison, recruitment of transcription factors is much more reliable to be detected with ChIP-PCR or ChIP-Seq assay, which may serve as a better reporter. In our study, GLIS2 knockdown caused H3K27ac increase on *PUMA* enhancer, accompanied by increased p53 recruitment and *PUMA* expression. These demonstrated that GLIS2 functions as a repressor of *PUMA* enhancer. Our data further showed GLIS2 regulates enhancer activity globally, probably through transcriptionally modulating p300. However, we do not exclude the possibility that GLIS2 may regulate p300 recruitment to enhancers via other mechanisms. A previous study reported that GLIS2 interacts with HDAC3 and regulates gene expression in several kidney cancer cells^34^. It is possible GLIS2 represses enhancer activity through removing H3K27ac. Unfortunately, we failed to reproduce the experiment in our system. Meanwhile, we did not detect the interaction between GLIS2 and p300. However, GLIS2 may still modulate enhancer activity through other mechanisms. In our study, we found that GLIS2 knockdown affects the p53 binding to PUMA, but not p21, which is consistent with the mRNA expression. It suggests that GLIS2 is possibly involved in the regulation of p53 recruitment, but the detailed mechanisms require further studies.

We found one dataset of eGFP-GLIS2 ChIP-Seq in HEK293 on ENCODE. Though the dataset supported the localization of GLIS2 on p53 target genes, but the exact GLIS2 binding sites was far away from p53 sites of our study. It was maybe due to the different distribution of GLIS2 in different cells. So, we performed Capture-ChIP with HCT116 cells stable expressing FB-tagged GLIS2, which showed nice co-localization of GLIS2 with p53. Our data will be also useful to study the function of GLIS2 in other pathways.

### Conclusions

To sum up, our study proved that GLIS2 acts as an oncogene in colorectal cancer repressing apoptosis and promoting cell migration. It transcriptionally represses the expression of the apoptotic gene *PUMA* and genes related with focal adhesion. GLIS2 regulates transcription through repressing the expression of p300 and the activity of enhancers, including H3K27ac inhibition and prevention the binding of transcription factors. Taken together, we have proved GLIS2 as an oncogene in colon cancer through selectively regulating gene transcription and enhancer activity.

## Supplementary information

Supplemental Figures

Supplemental datasets

## Data Availability

All the deep sequencing data have been submitted to GEO database, with the Acc. NO. GSE125928.
