# Supplementary material for: GLIS2 promotes colorectal cancer through repressing enhancer activation
Source: Oncogenesis. 2020 Jun 1;9(5):57. doi: 10.1038/s41389-020-0240-1 (PMC7264249; doi:10.1038/s41389-020-0240-1)
Supplement: Supplementary file 1 — Supplemental Figures [file 41389_2020_240_MOESM1_ESM.pdf]

Sup. Fig. 1

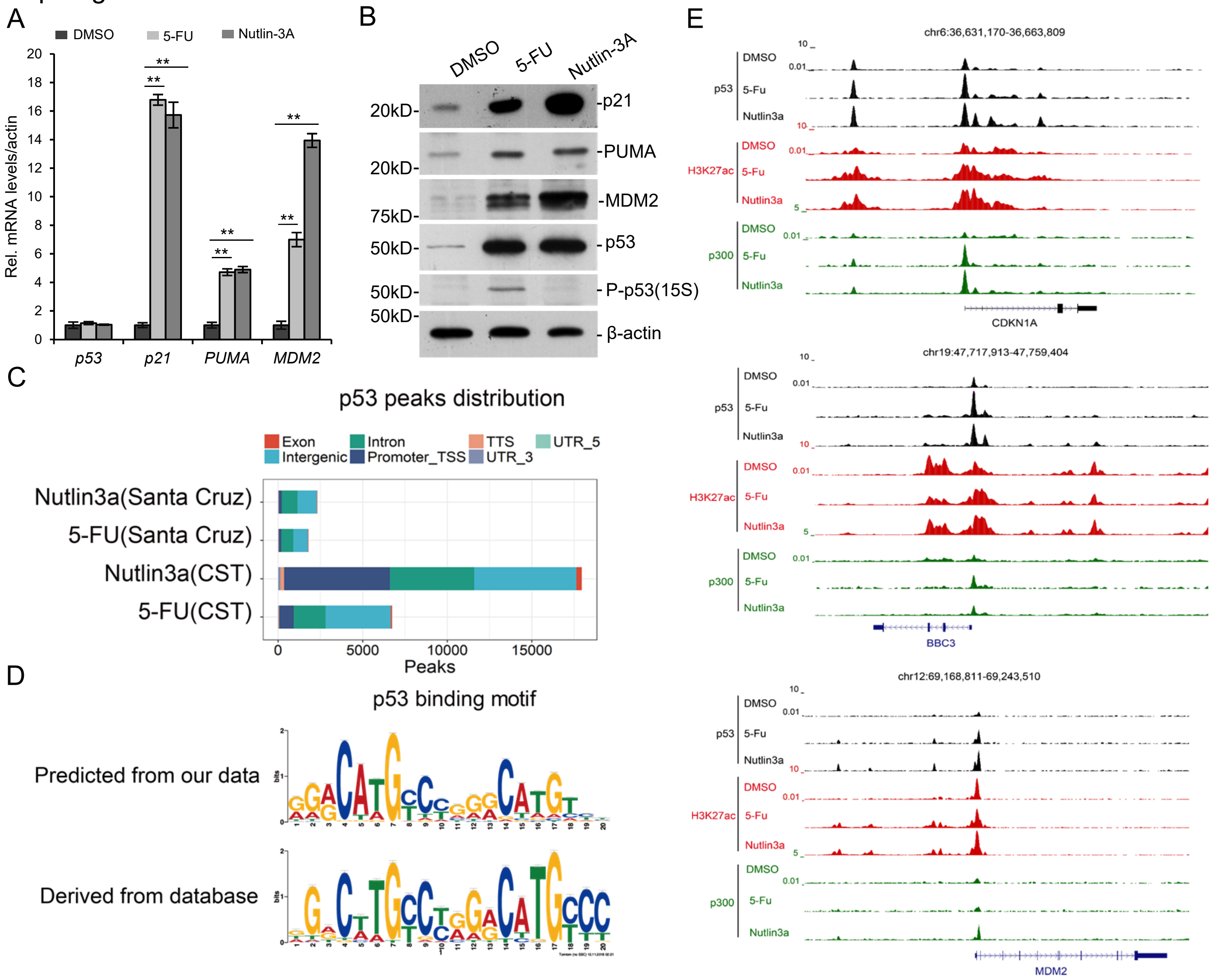

Sup. Fig. S1. Analysis of p53 ChIP-seq. (A&B) HCT116 cells were treated with 375μM 5-FU and 10μM Nutlin3a for 8 h. The expression of p53, p21, PUMA and MDM2 was determined by qRT-PCR(A) and western blotting(B). **(C)** Genome-wide p53 distribution pattern on different genome elements under DMSO, 5Fu and Nutlin3A treatment. **(D)** Predicted (upper) and derived (lower) consensus sequence motifs in the p53-binding regions. **(E)** The UCSC browser view shows p53, H3K27ac and p300 enrichment around CDKN1A (p21), BBC3 (PUMA) and MDM2. * p-value＜=0.05， ** p-value＜=0.01. (t-test). Histograms are presented as mean ± s.d. of three biological replicates.

Sup. Fig. 2

A

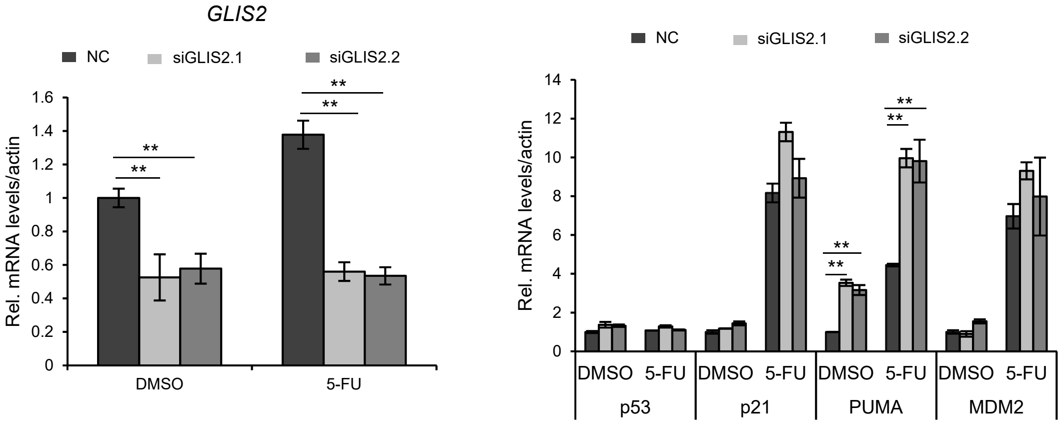

B

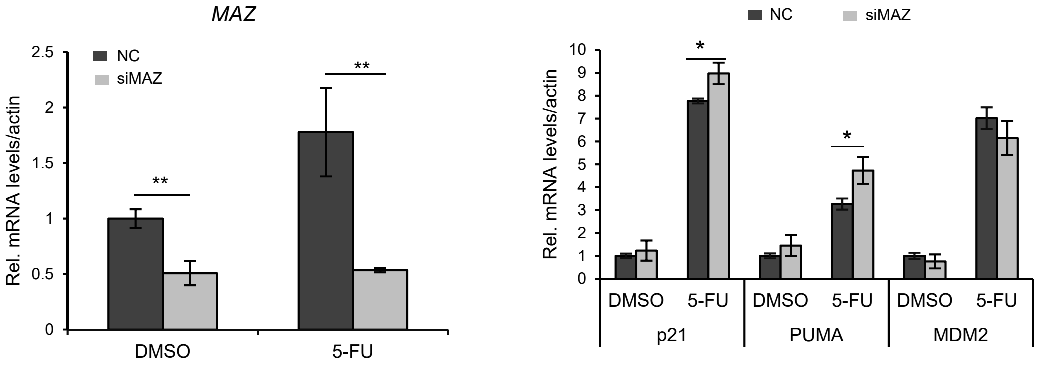

C

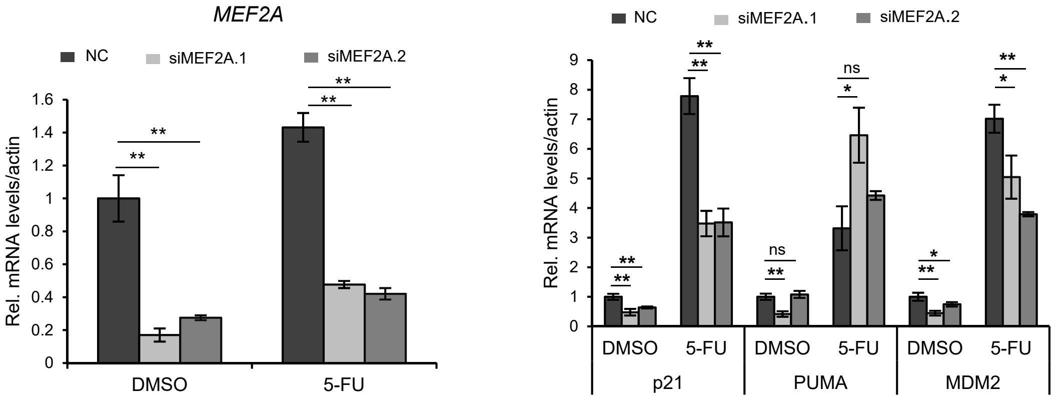

Sup. Fig. S2. Screening of p53 co-regulators. (A-C) siRNAs targeting predicted p53 co-regulators MAZ(A), MEF2A(B) and GLIS2(C) were transfected into HCT116. 48 h after transfection, 375μM 5-FU was withdrawn, and 8 h later, the expression of PUMA, p21 and MDM2 was determined by qRT-PCR. * p-value＜=0.05， ** p-value＜=0.01. (t-test). Histograms are presented as mean ± s.d. of three biological replicates.

Sup. Fig. 3

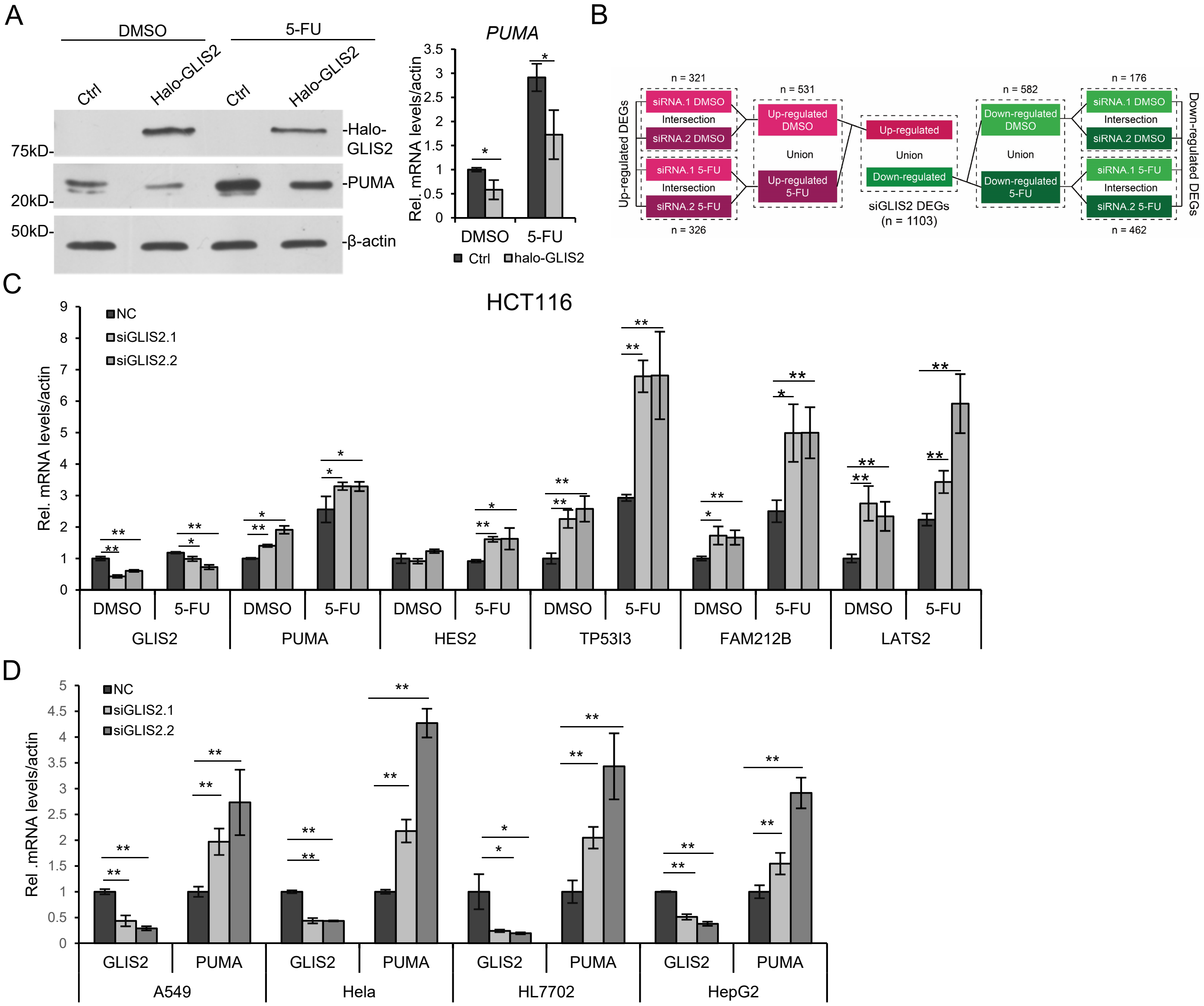

Sup. Fig. S3. GLIS2 selectively regulates the expression of p53 target genes. (A) Halo-GLIS2 stably expressed cells were performed for western blotting and qRT-PCR to determine the expression of PUMA. **(B)** A sketch for the pipeline of DEGs analysis after GLIS2 knockdown. **(C)** Validation of the expression of some p53 target genes after GLIS2 knockdown by qRT-PCR. **(D)** GLIS2 knockdown increased the activation of PUMA in A549, Hela, HL7702 and HepG2. * p-value$\leq$=0.05， ** p-value$\leq$=0.01. (t-test). Histograms are presented as mean ± s.d. of three biological replicates.

Sup. Fig. 4

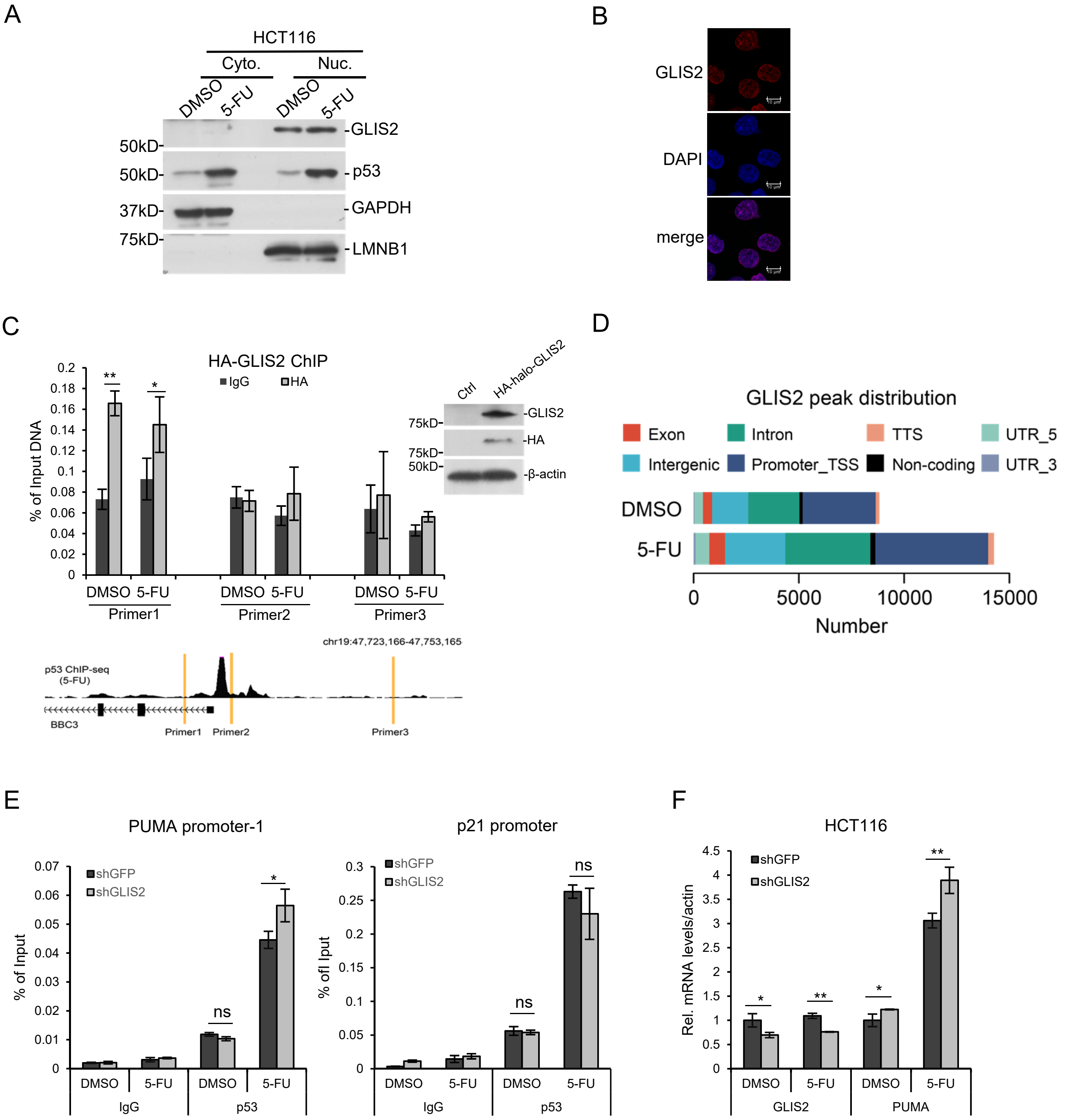

Sup. Fig. S4. GLIS2 represses p53 recruitment to PUMA. (A) HCT116 cells were treated with 375μM 5-FU for 8 hr. Cytoplasmic and nuclear fractions were prepared and immunoblotted with the indicated antibodies. **(B)** Immunofluorescent microscopy was performed in HCT116. **(C)** HA-GLIS2 stably expressed cells were performed for HA ChIP assay. Data show GLIS2 binding on PUMA relative to IgG. The primer sets for qPCR are indicated at the bottom. The inset shows a western blot of the GLIS2 level in the stable cell line. **(D)** The genome distribution of GLIS2 peaks in the control and 5-Fu-treated HCT116 cells. **(E)** ChIP analysis shows p53 enrichment on PUMA and p21 promoters in HCT116 shGFP and shGLIS2 stable cell lines. **(F)** qRT-PCR shows GLIS2 and PUMA expression in HCT116 shGFP and shGLIS2 stable cell lines. * p-value＜=0.05， ** p-value＜=0.01. (t-test). Histograms are presented as mean ± s.d. of three biological replicates.

Sup. Fig. 5

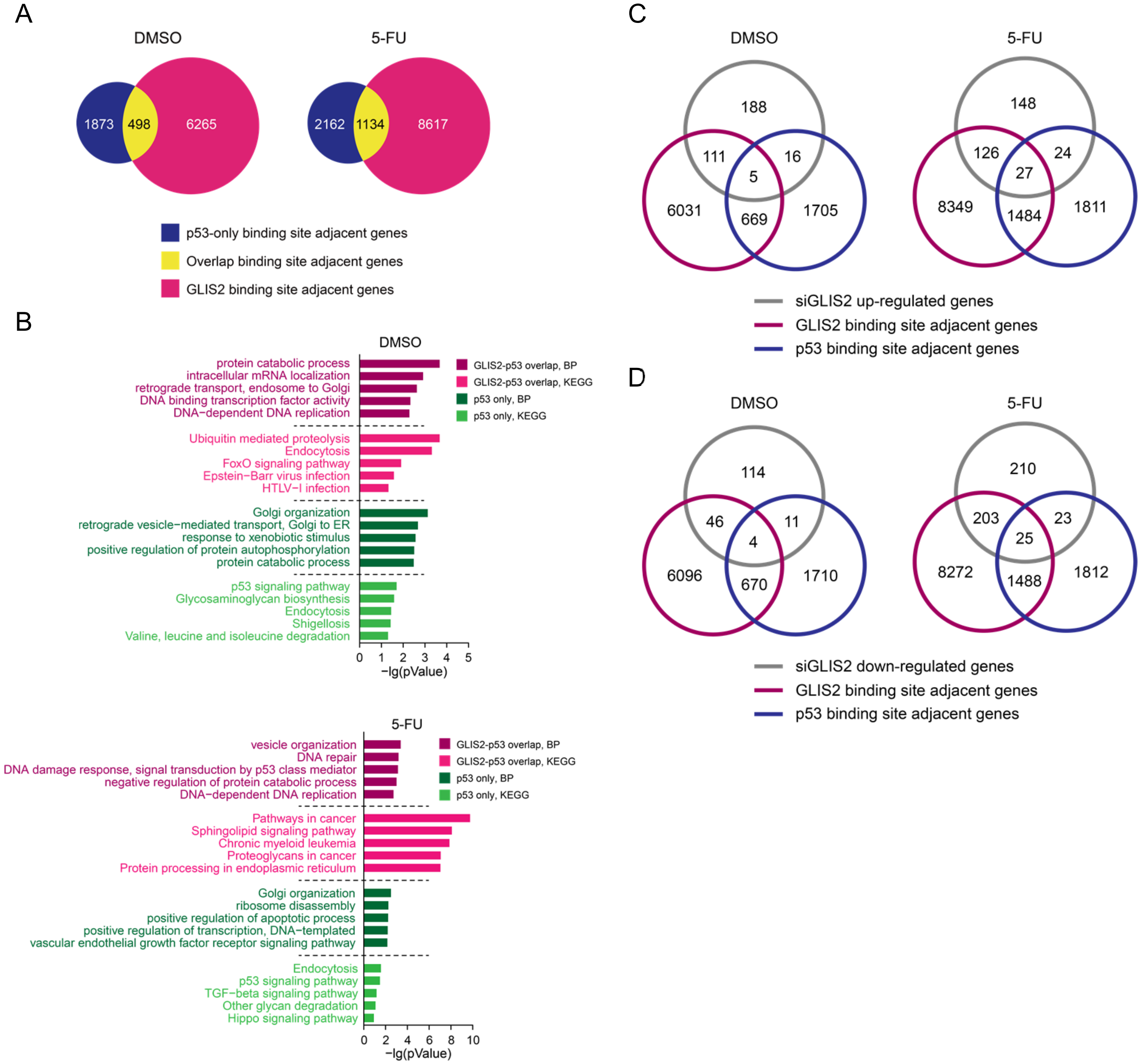

Sup. Fig. S5 The comparision of GLIS2 and p53 binding on chromatin. (A) Venn diagram shows the adjacent genes to p53 unique, GLIS2 unique and their overlapped sites. If two sites bound by the two proteins are within 200 bp, then they are considered as overlapped sites. **(B)** The enriched KEGG pathways and biological process analysis of the indicated gene groups in (A). **(C & D)** Venn Diagram shows overlapped genes between the GLIS2 regulated DEGs and p53 or GLIS2 binding genes.

Sup. Fig. 6

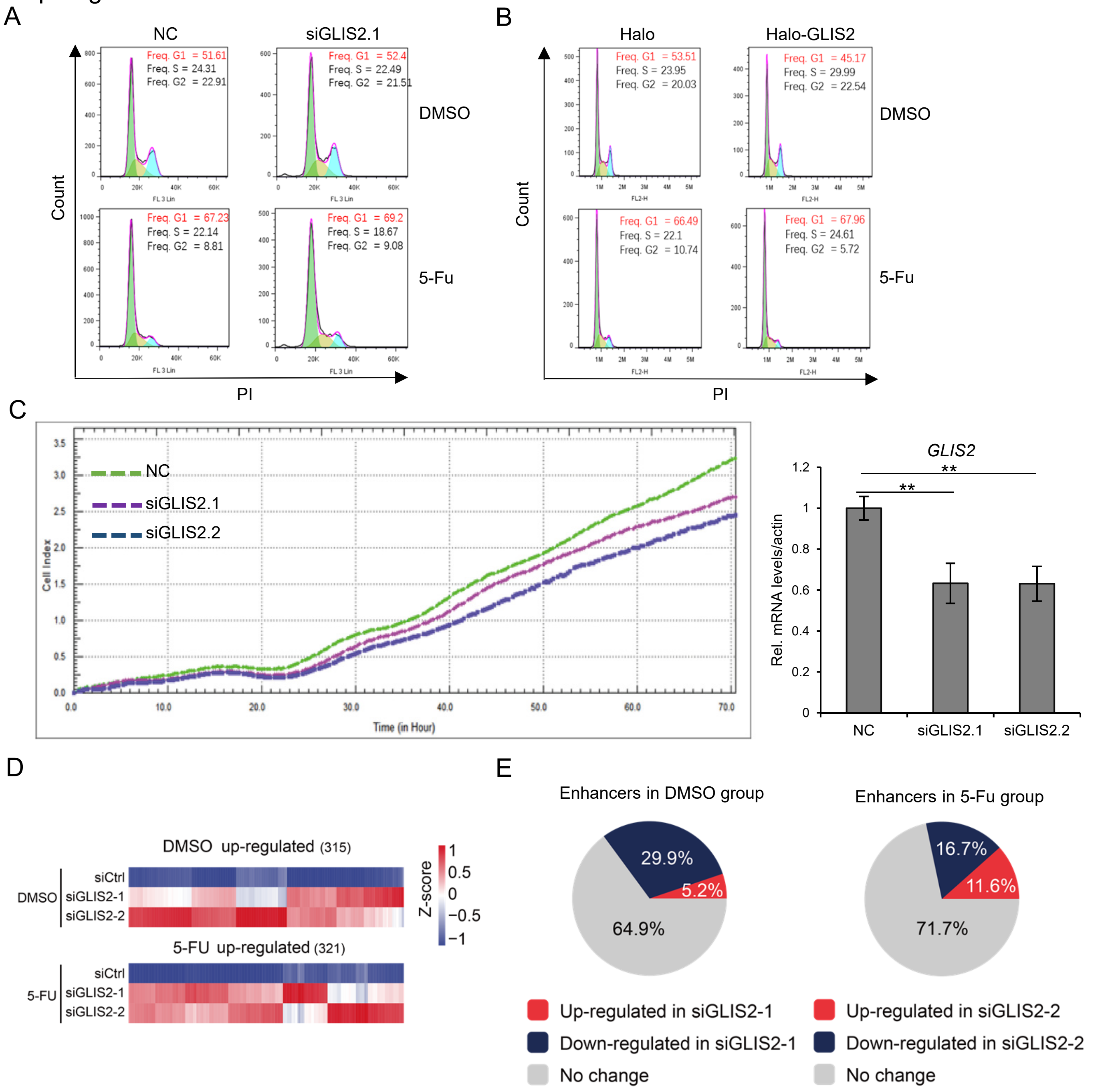

Sup. Fig. S6. GLIS2 promotes colon cancer cell proliferation. (A & B) Cell cycle was measured with propidium iodide (PI) staining followed by flow cytometry. **(C)** GLIS2 was knocked down with siRNAs in HCT116. The real-time cell proliferation was measured with RTCA assay according to the manufacturer's protocol. **(D)** Heat maps show the expression (FPKM) of the genes repressed by GLIS2. **(E)** The pie chart shows the percentages of H3K27ac changes on genome-wide enhancers after GLIS2 knockdown. * p-value＜=0.05， ** p-value＜=0.01. (t-test). Histograms are presented as mean ± s.d. of three biological replicates.

Sup. Fig. 7

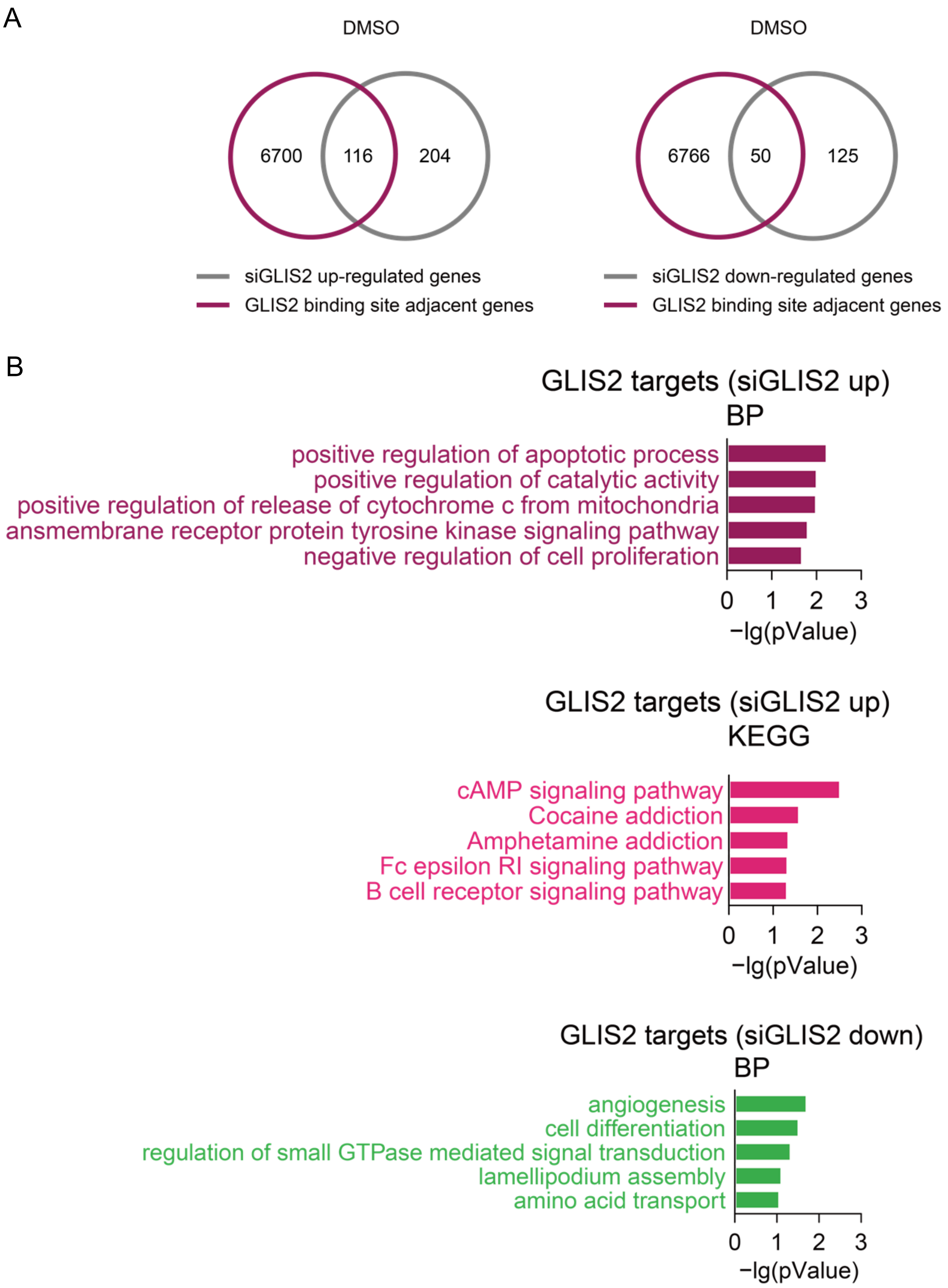

Sup. Fig. S7 Analysis of GLIS2 target genes. (A) Venn Diagram shows overlapped genes between the GLIS2 regulated DEGs and GLIS2 binding genes. **(B)** The KEGG pathways enrichment and biological process analysis of indicated gene clusters.
